# Optimising adherence to secondary prevention medications following acute coronary syndrome utilising telehealth cardiology pharmacist clinics: a matched cohort study

**DOI:** 10.1007/s11096-023-01562-4

**Published:** 2023-03-20

**Authors:** Adam C. Livori, Derk Pol, Bianca Levkovich, Ernesto Oqueli

**Affiliations:** 1 Pharmacy Department, Grampians Health Ballarat, 1 Drummond St Nth, Ballarat, VIC 3350 Australia; 2 Centre for Medicine Use and Safety, Monash University, Clayton, VIC Australia; 3 Monash Heart, Clayton, VIC Australia; 4 Latrobe Regional Hospital, Traralgon, VIC Australia; 5 School of Medicine, Faculty of Health, Deakin University, Geelong, VIC Australia

**Keywords:** Acute coronary syndrome, Cardiology, Medication adherence, Percutaneous coronary intervention, Pharmacists

## Abstract

**Background:**

Adherence to secondary prevention medications following acute coronary syndromes (ACS) is a predictor of future major adverse cardiovascular events. Underutilisation of these medications is associated with higher risk of major adverse cardiovascular events globally.

**Aim:**

To explore the effects of a telehealth cardiology pharmacist clinic on patient adherence to secondary prevention medications in the 12 months following ACS.

**Method:**

Retrospective matched cohort study within a large regional health service comparing patient populations before and after implementation of pharmacist clinic with 12-month follow up. Patients who received percutaneous coronary intervention for ACS were consulted by the pharmacist at 1, 3- and 12-months. Matching criteria included age, sex, presence of left ventricular dysfunction and ACS type. Primary outcome was difference in adherence in adherence at 12 months post ACS. Secondary outcomes included major adverse cardiovascular events at 12 months and validation of self-reported adherence using medication possession ratios from pharmacy dispensing records.

**Results:**

There were 156 patients in this study (78 matched pairs). Analysis of adherence at 12 months demonstrated an absolute increase in adherence by 13% (31 vs. 44%, *p* = 0.038). Furthermore, sub-optimal medical therapy (less than 3 ACS medication groups at 12 months) reduced by 23% (31 vs. 8%, *p* = 0.004).

**Conclusion:**

This novel intervention significantly improved adherence to secondary prevention medications at 12 months, a demonstrated contributor to clinical outcomes. Primary and secondary outcomes in the intervention group were both statistically significant. Pharmacist-led follow up improves adherence and patient outcomes.

## Impact statements

- Implementing cardiology pharmacist telehealth clinics leads to improved medication adherence, and by extension improved clinical outcomes in patients following an acute coronary syndrome.
- This model of care is now a permanent component of care for patients following discharge for an acute coronary syndrome across a large regional health service.
- This model is now being adapted in a rapid access atrial fibrillation clinic model to enhance anticoagulant and anti-arrhythmic management.

## Introduction

Cardiovascular disease represents a major burden on health worldwide. In 2016, 43,963 deaths were attributed to heart disease nationally, equating to 30% of all deaths [1]. The number one cause of cardiovascular disease death and morbidity is acute coronary syndromes (ACS) [1]. Developed parts of the world all share in the burden of cardiovascular disease, with similar rates of mortality and morbidity across Europe, North America and Australia [1]. In Australia, due to its large degree of urbanisation, health disparities exist for regional Australians versus their metropolitan counterparts, where there is a higher rate of early deaths from cardiovascular disease compared to metropolitan areas [2].

Advances in access to catheter laboratories and stent technology have all contributed to increased survival following acute coronary syndrome (ACS) events [3]. However, medications remain the mainstay of treatment for secondary prevention of subsequent events [3–5]. In particular, there are a number of medications directly linked with mortality benefit and reduction of major adverse cardiovascular events (MACE). These medications are referred to as optimal medical therapy (OMT) and consist of four groups: dual anti-antiplatelet therapy (DAPT), which includes aspirin and an adenosine diphosphate (P2Y12) inhibitor (clopidogrel, prasugrel, or ticagrelor); HMG Co-A reductase inhibitors (statins); beta blockers; and angiotensin converting enzyme inhibitors/angiotensin receptor blockers (ACEI/ARB). More recently, angiotensin receptor and neprilysin inhibitors (ARNI) have also been used in place of ACEI in patients with pre-existing heart failure with reduced ejection fraction (HFrEF) [6].

While the randomised control trials (RCT) that have generated clinical outcomes data show that the presence of these medications reduces MACE, the conditions of those trials are not necessarily representative of real-world populations [5]. For example, real-world statin studies often show non-adherence rates from 10 to 45%, with RCT data showing non-adherence rates of 1–2% [7, 8]. These observational, real-world studies provide insight into how these therapies are utilised within the population. A multicentre registry study of 19,704 patients in the United States of America showed that at 90 days post ACS, mean adherence to DAPT was 72%, beta blockers 63%, statin 63% and ACEI 64% [9]. The link between adherence to therapy and clinical outcomes has also been researched extensively within the ACS population. An Australian registry study of 9,735 patients demonstrated that being on less than three secondary prevention medications (sub-optimal medical therapy) was an independent predictor of long-term mortality at 4 years, when compared to being on all OMT medications (8.2% vs. 16.8%, *p* < 0.001, n = 9375) [3].

There are many factors associated with poor medication adherence, such as socioeconomic status, age, and health literacy [10, 11]. These aspects are also strong determinants of multi-morbidity and cardiovascular outcomes, with multidisciplinary interventions recommended to optimise outcomes [12]. Poor understanding of medications is a contributor to poor medication adherence, illustrating the importance of providing appropriate medication and disease education to patients [11]. In an American study of 5014 statin users, only 1654 (33%) were aware of why they were prescribed a statin and what relevance it had to their cardiovascular health [13]. Telehealth, the use of telephony and video-conferencing for communicating health, has been utilised by pharmacists in ambulatory care and cardiology for many years, using a variety of interventions and impact measures [14].

Telehealth is an established way of increasing services without affecting the quality of care. It has previously been utilised successfully in the management of cardiovascular diseases [15, 16]. The emerging requirement for non-patient facing consultations due to the COVID-19 pandemic also highlighted the need for telehealth-based interventions. There is evidence for the impact that a pharmacist can have as a part of the multi-disciplinary team providing care to patients in the ambulatory care setting [17–20]. One randomised control trial from Canada utilised pharmacists and telehealth, and involved phone calls of 5–10 min at 1 week, 1 month, 6 months and 9 months post coronary stenting. The data illustrated that at 12 months 87.2% (n = 131/150) of patients in the intervention arm remained on clopidogrel with only 43.1% (n = 65/150) of patients in the control (no phone call follow up) arm [21]. This study seeks to build upon existing literature in targeted medication adherence interventions [22].

### Aim

This study aimed to explore the effects of a telehealth cardiology pharmacist clinic on patient adherence to secondary prevention medications in the 12 months following ACS.

### Ethics approval

This study was granted approval and waiver of consent by Grampians Health Ballarat Human Research Ethics Committee (Project number LNR/71907/BHSSJOG) and Monash University (28159).

## Method

### Study design

This was a retrospective matched cohort study with a 12 month follow up duration from the index PCI. It utilised an existing data source of patients who received PCI with coronary stenting. The study consisted of two groups, an intervention group from 2020 who received a consult in the telehealth cardiology pharmacist clinic, and a control group of patients prior to 2020 who did not receive the intervention.

### Telehealth cardiology pharmacist clinic

The telehealth cardiology pharmacist clinic involves a 20-min consultation with a pharmacist addressing a number of different care elements. The mode of delivery of the service, as well as patient and clinician acceptability has been assessed in a previous study [23]. A detailed best-possible medication history is taken with secondary source verification in line with the Society of Hospital Pharmacists Australia: Standards of Practice in Clinical Pharmacy [24, 25]. Questions regarding cardiac symptoms and a functional assessment are completed, which align with the outcome measures defined in the Melbourne Interventionalist Group (MIG) assessment form. These questions are designed to understand the burden of cardiac symptoms as well as identifying any potential urgent referral points for patients. Education on cardiac health and treatment is provided in line with the National Heart Foundation of Australia National Cardiac Rehabilitation Quality Indicators [26]. Patients are consulted at 1 month, 3 months and 12 months post PCI. The format is repeated for each of the consultations, with a focus on building patient knowledge of cardiac medications and health. When barriers to access of medications are noted, the pharmacist took steps to rectify this by consultation with the cardiology unit and/or the primary care physician. Examples would be arranging new prescriptions, recommending modification of dose or dosage forms, and implementing dose administration aids. Additionally, attendance at cardiac rehab was assessed and referrals made where indicated.

### Study setting

The study was based at a large public regional health service in Victoria, utilising data collected as part of the health services participation in the Victorian Cardiac Outcomes Registry (VCOR) and MIG, as well as data from the telehealth cardiology pharmacist clinic consult letters.

### Study participants

There are approximately 250 PCI and coronary stent patients who present to Grampians Health Ballarat annually with ACS. Patients who received PCI were enrolled into an opt-out MIG registry. Data are collected at the time of PCI and at 30 days. Twelve-month follow up data were available for patients who received PCI with coronary stents in the 2015–2017 cohort. From 2017, routine 12 month follow up data were not collected.

Patients in the intervention arm had their baseline MIG data collected at time of PCI and at 30 days. Due to the lack of 12-month MIG data, the telehealth cardiology pharmacist clinic report was used to check if OMT was present at 12 months.

In order to allow for comparison between control and intervention groups, set criteria were established to ensure 12-month follow up data were available. It was the intention of this study to enrol all patients who received PCI for ACS during the study period of January 2020 to July 2020.

Inclusion criteria:

- Adults aged 18 years or older, no upper age cut-off
- Diagnosed with ACS requiring PCI with coronary stenting, inclusive of
  - ST elevation Myocardial Infarction (STEMI)
  - Non-ST elevation Myocardial infarction (NSTEMI)
  - Unstable angina
- Patients who had 12-month follow up data available for analysis (either via registry or clinic for control and intervention arms respectively)
- Patient or carer able to participate in telehealth consult or via phone (intervention arm only).

Exclusion criteria:

- Patients with ACS not treated with PCI who were transferred for surgical intervention
- PCI without stent deployment (balloon angioplasty only)
- Elective PCI for stable angina
- Patients with unsuccessful PCI that were escalated to surgical management
- Patients who chose to opt-out from registry or clinic at any time during the 12-month follow up period.

### Adherence definitions

Adherence to medication classes was broken up into three groups, similar to previous studies [3, 27]. These groups included:

- Optimal Medical Therapy (OMT) (all four medication groups)
- Near-optimal Medical Therapy (NMT) (three medication groups)
- Sub-optimal Medical Therapy (SMT) (less than three medication groups).

Patients are specifically asked about adherence to each medication comprising OMT, with adherence defined as taking the medication more than 80% of the time [28].

### Study outcomes

The primary outcome was the difference of self-reported adherence to all four groups of secondary prevention medications (optimal medication therapy) at 12 months post coronary stenting between a matched cohort of patients who received the intervention and those who did not. Self-reported adherence was determined by a set assessment form by the Melbourne Interventionalists Group, where the pharmacist would ask the patient directly during the telehealth consultation. This questionnaire was identical between the control and intervention groups. Secondary outcomes included the difference in Near-optimal Medical Therapy and Sub-optimal Medical Therapy, and individual medication groups (DAPT, statin, beta blocker and ACEI/ARB/ARNI).

Additionally, the difference in Major Adverse Cardiovascular Events (MACE) at 12 months between control and intervention matched cohort was investigated. MACE was defined as stroke, non-fatal myocardial infarction, rehospitalisation or death. To validate the use of self-reported adherence within the study, self-reported adherence was compared to calculated medication possession ratio (MPR) via the patient’s primary pharmacy dispensing records. This outcome was only measured in the intervention group due to dispensing data availability.

### Statistical analysis

Cohort matching was used to reduce potential confounding between the control and intervention arms [29]. As this study is retrospective and non-randomised, the use of individual matching between cohorts provides a method to reduce confounding [29]. The matching criteria were selected due to both their availability within the data set and evidence regarding their significant correlation with changing adherence patterns between participants [30–32].

In this study, matching was performed using individual matching across criteria:

- Age stratification at time of percutaneous coronary intervention (PCI) [3]
  o < 50, 50–59, 60–69, 70–79, 80–89, > 89.
- Sex
  o Male, female.
- Type of acute coronary syndrome (ACS)
  o STEMI, NSTEMI, unstable angina.
- Left ventricular dysfunction at PCI defined as by stratified ejection fraction [33]
  o < 50%, ≥ 50%.

Data matching was indexed at the time of the ACS event and baseline MIG data collected. Matching and analysis was performed using Stata^®^ 17 and Microsoft Excel^®.^

Based on previous studies, adherence to medications at 12 months post ACS event can vary between 45 and 75% [3, 8]. Based on a population size of approximately 100 patients to have ACS in the 7-month intervention period (January 2020 to July 2020), a margin of error of 5% with an alpha of 0.05 and beta of 0.2, the sample size would need to be 73–78 matched patient pairs (one to one matching).

For outcome calculations, McNemar’s Chi-squared analysis for matched data was used between the control and intervention pairs. This was repeated across the primary and secondary outcomes involving matched data. For the adherence measure validation outcome, an R^2^ value was calculated between self-reported adherence scores and calculated MPR. A value of 0.75 or greater was considered a substantial correlation.

## Results

**Fig. 1:**
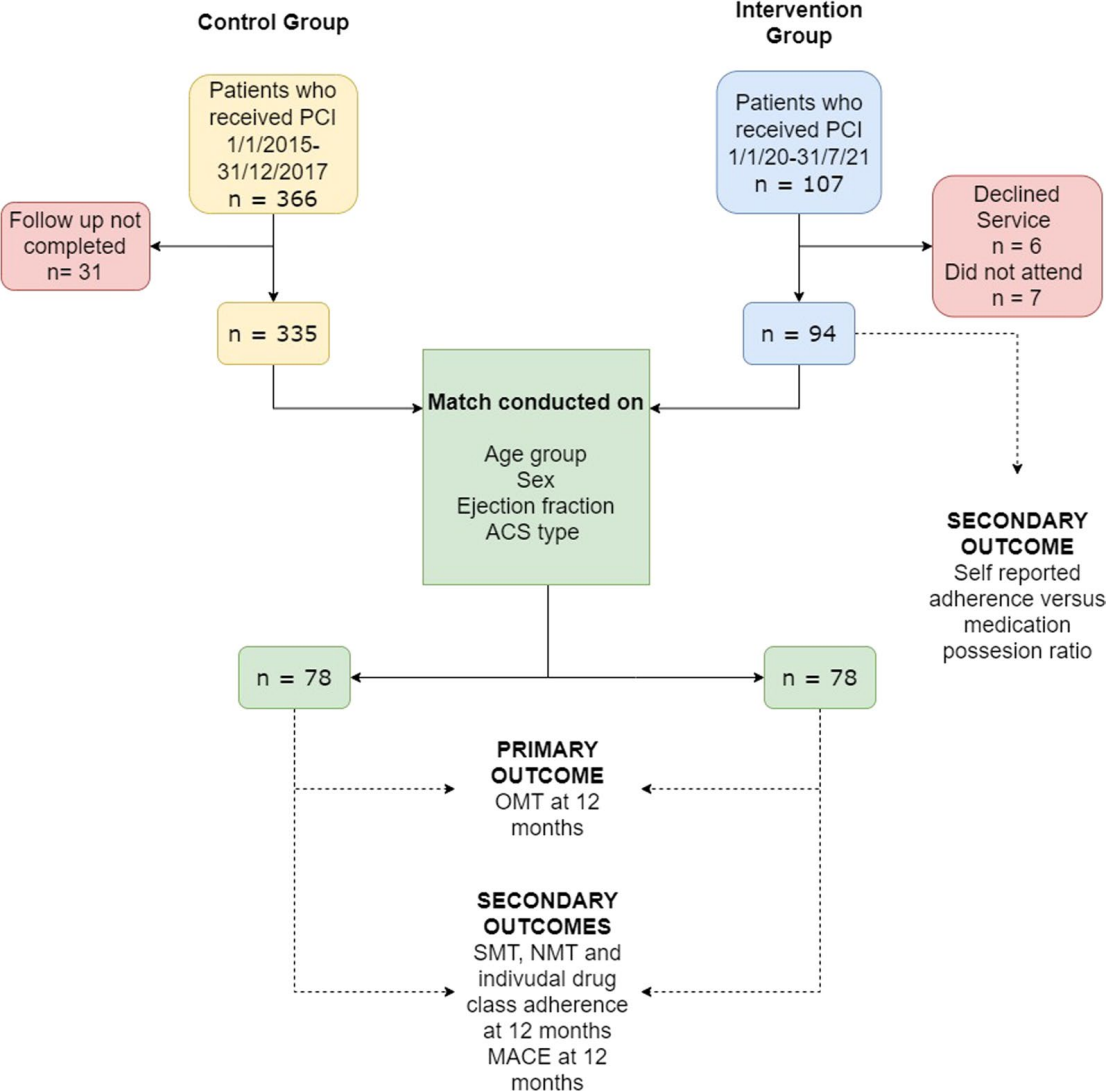
CONSORT diagram of participant selection, matching and analysis. *PCI* percutaneous coronary intervention, *ACS* Acute coronary syndrome, *OMT* optimal medical therapy, *NMT* near optimal medical therapy, *SMT* sub-optimal medical therapy, *MACE* major adverse cardiovascular event

The control cohort consisted of 366 patients in total across the 3-year period, with 335 patients having 12-month follow up data available for analysis. The intervention group contained 107 patients, thirteen of which were excluded as they declined the service, or did not attend any clinic sessions. From these two data sources, 156 patients (78 matched pairs) were matched using the pre-specified criteria detailed in the methods (Fig. 1). Following matching, there were no statistically significant differences in the demographic data available from MIG (Table 1).

**Table 1:** Characteristics of matched participants

	Control (n = 78)	Intervention (n = 78)	*p* value
Median age (inter-quartile range)	64 (56–71)	64 (58–71)	1.000
Male (%)	62 (84)	62 (84)	1.000
Smoking status (n, %)			
Smoker	25 (32)	17 (23)	0.080
Ex-smoker	27 (36)	37 (46)	0.150
Never smoked	25 (32)	23 (31)	0.880
Unknown	0 (0)	0 (0)	1.000
Diabetes (n, %)	16 (22)	16 (22)	1.000
Hypertension (n, %)	48 (65)	53 (72)	0.290
Previous MI (n, %)	12 (16)	15 (19)	0.580
Ejection fraction (n, %)			
> 50%	55 (74)	55 (74)	1.000
45–50%	7 (9)	7 (9)	1.000
35–44%	13 (18)	13 (18)	1.000
< 35%	3 (4)	3 (4)	1.000
Unknown	0 (0)	0 (0)	1.000
PCI Indication (n, %)			
STEMI	39 (53)	39 (53)	N/A
NSTEMI	36 (49)	36 (49)	N/A
Unstable angina	3 (4)	3 (4)	N/A
Rhythm (n, %)			
AF	6 (8)	6 (8)	1.000
Sinus	71 (96)	70 (95)	0.730
Other	1 (1)	2 (3)	0.310

*MI* myocardial infarction, LVEF left ventricular ejection fraction, *PCI* percutaneous coronary intervention, STEMI ST-elevation myocardial infarction, *NSTEMI* Non-ST-elevation myocardial infarction, AF atrial fibrillation

### Primary outcome

There was a significant difference in the number of patients on OMT at 12 months between the groups, in favour of the telehealth cardiology pharmacist clinic cohort. There were 78 matched pairs in the analysis of the primary outcome, with 24 of 78 (31%) patients adherent to OMT at 12 months post PCI in the control group and 34 of 78 (44%) patients in the intervention group demonstrating an absolute difference of 13% (*p* = 0.038).

### Secondary outcome

There was no statistically significant difference when comparing matched pairs of participants with near-optimal medical therapy (three out of four post ACS mediation groups present). However, there was a statistically significant absolute reduction in patients with sub-optimal medical therapy (less than three post ACS medication groups present) of 16% (33/78 vs 20/78, *p* = 0.04). Across each individual medication group, significant differences were seen between matched pairs across all classes except for beta blockers (Table 2).

**Table 2:** Changes to medical therapy and individual therapy adherence at 12 months for matched participants

	Control n = 78	%	Intervention n = 78	%	*p* value
Adherence to optimal medical therapy at 12 months (all four groups presentation)	24	31	34	44	0.038
Near-optimal medical therapy at 12 months (three groups present)	21	27	24	31	0.719
Sub-optimal medical therapy at 12 months (less than three groups present)	33	42	20	26	0.035
DAPT at 12 months	44	56	60	77	< 0.010
Statin at 12 months	65	83	67	86	0.010
Beta blocker at 12 months	54	69	66	85	0.790
ARB/ACEI/ARNI at 12 months	44	56	60	77	< 0.010

*DAPT* dual anti-platelet therapy, *ARB* angiotensin receptor blocker, *ACEI* angiotensin converting enzyme inhibitor, *ARNI* angiotensin receptor neprilysin inhibitor

There was a significant reduction in major adverse cardiovascular events (MACE) consisting of a 4-point composite outcome of stroke, non-fatal myocardial infarction, rehospitalisation or death. The was an absolute reduction of MACE of 22% (34/78 vs. 17/78, *p* < 0.01). This was driven primarily through a reduction in hospitalisations (Table 3).

**Table 3:** Major adverse cardiac events (MACE) at 12 months for matched participants

	Control n = 78	%	Intervention N = 78	%	*p* value
Major adverse cardiovascular event	34	44	17	22%	0.004
Stroke	0	0	0	0	–
Non-fatal myocardial infarction	4	5	3	4	–
Rehospitalisation	31	40	15	19	–
Death	2	3	2	3	–

**Fig. 2:**
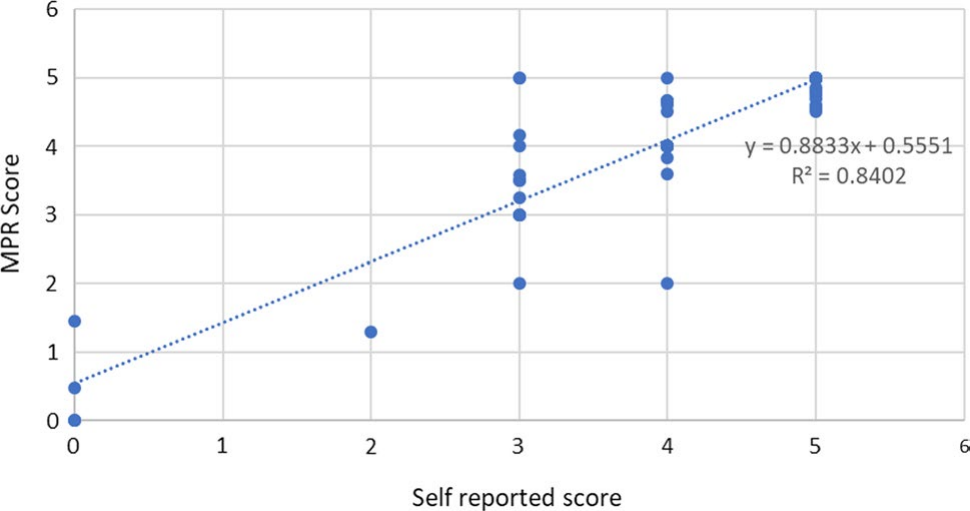
Self-reported adherence to medical therapy versus medication possession ratio (MPR)

For the validation of self-reported adherence within the study population, all intervention group participants (n = 98) were included. This outcome compared the self-reported adherence scores to the medication possession ratio (MPR) sourced from participant dispensing histories. The linear regression model demonstrated a R^2^ value of 0.84, with 17% (16/94) of participants underestimating and 13% (12/94) participants overestimating adherence when compared to MPR. The remaining 70% (66/94) of participants had MPR within 10% of the self-reported adherence (Fig. 2).

## Discussion

This matched retrospective cohort study demonstrated a 13% absolute increase in the degree of adherence to Optimal Medical Therapy (OMT) at 12 months post an acute coronary syndrome (ACS) event. Furthermore, a significant decrease in patients with sub-optimal adherence to medical therapy was observed, which may translate into further reductions in MACE as per previously published work utilising the state-wide MIG registry [3].

The analysis of individual medication groups demonstrate increases in adherence at 12 months when comparing matched pairs, with the exception of beta blockers. Given the trend of other therapies, one possible explanation of why beta blockers did not see an increase was due to a change in recommendations of beta blockers in Non-ST-Elevated Myocardial Infarction (NSTEMI). There has been discussion in guidelines regarding the role of beta blockers in NSTEMI with revascularisation, where beta blockers are no longer recommended in the absence of left ventricular systolic dysfunction [34]. This study was not powered to investigate these individual relationships between ACS types.

The significant reduction in MACE was driven primarily by hospital admissions, with a substantial increase in the risk of readmissions in the control group. When considering secondary prevention medications and reductions in MACE, signs of benefit are not fully detected until after 12 months in this setting [3].

The use of self-reported adherence is easy to ascertain from a cost and time perspective, but is challenged with the balance between non-adherence and non-prescription [3]. This study’s use of a secondary outcome of internally validated self-reported adherence within this population provides proof of prescription and dispensing via the review of dispensing records independent to patient self-reporting. The use of two separate measures improves accuracy, particularly when a combination of subjective (self-reported adherence) and objective (medication possession ratio) measures are used [28]. Self-reported outcomes have been documented as being the least reliable, often associated with “white coat adherence” and overestimation [28]. However, the data from this study suggest that this population is just as likely to underestimate as they are to overestimate, with the majority of patients self-reporting within 10% of their calculated MPR.

### Limitations

Limitations in this study include its single-centre setting and although this represents the population of the area the health service operates, this may not reflect Australian or international populations. Telehealth has seen drastic uptake in the COVID-19 era, presenting itself with new challenges in the ambulatory care setting. However, this telehealth model of care was established and validated with patients and clinicians prior to the COVID-19 pandemic, and therefore was not overly affected by changes in practice [23].

Although this study does not replace a randomised study, it has employed various techniques to utilise the benefits randomisation brings to a study. While the matching variables selected have been shown to explain differences in adherence in previous studies, it does not possess the potential power of other methods such as propensity matching [35]. However, this study used population analysis rather than sampling, and randomisation does not control this variance as no sample is drawn. With the population sampled, total adherence within almost all possible recruitment was known. In addition, unmatched comorbidity characteristics showed little difference overall, and adjustment of these variables would have been unlikely to shift the outcome given the high degree of significance.

The decision to add therapies is directed by evidence-based guidelines, but also must involve tailoring to the patient’s individual needs and safety. This study treated absence of therapy as non-adherence, however contraindications to therapy or safety outcome guided cessation of therapies may have contributed to what was analysed as non-adherent. This is a common limitation of studies like this, and while the 12-month follow up was the point of interest, dynamic changes in patient medication prescription are not present in the data [3]. However, regardless of therapies prescribed at discharge, the intervention group saw an increase in medication adherence at the end of the follow-up relative to those who did not receive the intervention.

## Conclusion

This study demonstrated that for patients with acute coronary syndromes, a telehealth cardiology pharmacist clinic added to standard care was able to improve adherence to secondary prevention medications at 12 months. The cardiology pharmacist telehealth clinic increased individual adherence to all drug classes with the exception of beta blockers, and was associated with reduced MACE in the first 12 months following an acute coronary syndrome. This model of care has become a permanent service within Grampians Health, and is being translated into a pharmacist-physician model of care focusing on rapid access atrial fibrillation clinics to improve patient care and utilisation of anticoagulants and anti-arrhythmic agents.
